# Mechanism and Optimized Design Methodology of Steel Plate Reinforcement for Tunnel Lining Void Zones

**DOI:** 10.3390/ma18174204

**Published:** 2025-09-08

**Authors:** Shuai Shao, Yimin Wu, Helin Fu, Jiawei Zhang

**Affiliations:** 1 School of Hydraulic and Civil Engineering, Ludong University, Yantai 264025, China; 2 School of Civil Engineering, Central South University, Changsha 410075, China; fu.h.l@csu.edu.cn; 3 Tianjin Municipal Engineering Design & Research Institute, Tianjin 300051, China; zhangjw.112358@gmail.com

**Keywords:** tunnel lining void, steel plate reinforcement, numerical simulation, concrete damage plasticity (CDP) model, reinforcement spacing optimization, structural adhesive, chemical anchor bolt

## Abstract

Voids behind tunnel linings are common hidden defects in underground engineering, leading to reduced structural capacity and potential safety hazards. To address the deficiencies in the understanding of the mechanism and the optimization of design of the existing steel plate reinforcement methods, this study systematically investigates the reinforcement mechanisms and proposes refined design strategies through numerical simulations and experimental validation. First, a comparative analysis of the Concrete Damage Plasticity (CDP) model and the Extended Finite Element Method (XFEM) revealed that the CDP model exhibits superior accuracy and computational efficiency in simulating large-scale void linings. Second, the effectiveness of different reinforcement schemes (chemical anchor bolts alone, structural adhesive alone, and combined systems) was evaluated, demonstrating that structural adhesive dominates stress transfer, while chemical anchor bolts primarily prevent plate detachment. Through further optimization simulations of the steel plate spacing, it was found that a spacing of 0.25 m can balance the reinforcement effect and cost. This spacing restricts the maximum principal stress (1.83 MPa) below the tensile strength of concrete while essentially eliminating damage to the lower surface of the lining. An optimized steel plate reinforcement structure was ultimately proposed. By reducing the number of chemical anchor bolts and decreasing their size (with only M12 chemical anchor bolts arranged at the edges), local damage is minimized while maintaining reinforcement efficiency. The research results provide theoretical support and engineering guidance for the safe repair of tunnel void areas.

## 1. Introduction

Voids behind tunnel secondary linings represent a prevalent yet concealed defect in underground engineering, whose prolonged evolution may lead to reduced load-bearing capacity of lining structures, aggravated water leakage, and even sudden collapses [1,2,3]. Recent inspection data from numerous existing tunnels reveals that over 40% of operational tunnels contain undetected void-type defects. These hidden defects may progressively induce lining structure cracking and steel corrosion through multi-factor coupling effects including surrounding rock creep, groundwater erosion, and train vibrations [4,5,6]. Although various void treatment technologies have been proposed in academic research, the complexity of reinforcement mechanisms and practical constraints in engineering applications continue to hinder the improvement of remediation effectiveness [7,8].

The formation of secondary lining voids is primarily attributed to inadequate grouting during construction, differential surrounding rock deformation, and material shrinkage [2,9]. Research demonstrates that void-induced stress redistribution in linings can generate localized tensile stresses reaching 2.3 times the design value, significantly compromising structural durability [3,10]. More critically, conventional detection methods (e.g., hammer sounding, ground-penetrating radar) exhibit limited resolution and operational constraints, particularly in identifying small-scale voids (<5 cm) and characterizing their 3D spatial distribution [11,12]. A representative case involves a Shanghai metro tunnel that experienced sudden lining exfoliation after 10 years of operation, where a post-collapse investigation revealed undetected void zones during initial inspections [13]. Such incidents underscore both the technical deficiencies in existing detection methodologies and the long-term cumulative effects of void-related hazards [5,6].

Current mainstream void remediation approaches can be classified into three categories: grouting reinforcement, structural strengthening, and structural reconstruction. Grouting technology has gained widespread application due to its cost-effectiveness and adaptability, though its efficacy critically depends on grout diffusivity and interfacial bonding performance [14,15]. While cement-based grouts demonstrate high strength advantages, their practical implementation is constrained by poor flowability and hydration shrinkage issues, frequently leading to secondary void formation [16]. Polymer-based grouting materials (e.g., polyurethane) achieve rapid filling but exhibit significant durability degradation under chemical corrosion [17]. In recent years, micro-expansive concrete has also been widely used in the treatment of lining diseases [18,19]. However, it is difficult for the filled part to form a force-bearing whole with the original lining. In structural strengthening methods, carbon fiber-reinforced polymer (CFRP) sheets effectively inhibit crack propagation through tensile strength enhancement, yet demand exceptional construction precision [20]. The steel plate reinforcement system employs a composite bonding mechanism combining chemically bonded anchors and epoxy adhesives, achieving synergistic interaction between plates and linings that significantly improves stress redistribution characteristics in void-affected zones [21,22]. Experimental studies demonstrate 35–42% increases in ultimate bearing capacity when reinforcing crown voids exceeding 30 mm height [23]. Full-circumference steel plate reinforcement forms closed load-bearing systems through circumferential continuous welding, enhancing structural stiffness by 2.1–3.5 times [24,25]. The application of ultra-high-performance concrete (UHPC) as bonding layers further enhances interfacial shear resistance [25]. From process innovation perspectives, researchers have developed graded loading methods for steel plate prestressing application, achieving precise displacement control (0.1–0.3 mm) through hydraulic jack systems [26,27].

This study addresses the critical issues of insufficient mechanical mechanism understanding and lack of design optimization in current steel plate reinforcement methods for void linings. Guided by the principles of energy conservation, emission reduction, and cost-effectiveness enhancement, we systematically investigated the reinforcement effects of steel plate-strengthened void linings through numerical simulation. The field implementation scheme was subsequently optimized with particular emphasis on determining the optimal steel plate spacing. Furthermore, based on mechanistic analysis of the reinforcement mechanism, we improved the structural detailing configuration of steel plate reinforcement to enhance reinforcement efficiency while reducing rehabilitation costs. The findings provide both theoretical guidance and practical solutions for the performance enhancement of void lining structures.

## 2. Case Implementation of Steel Plate Reinforcement Scheme

**Figure 1:**
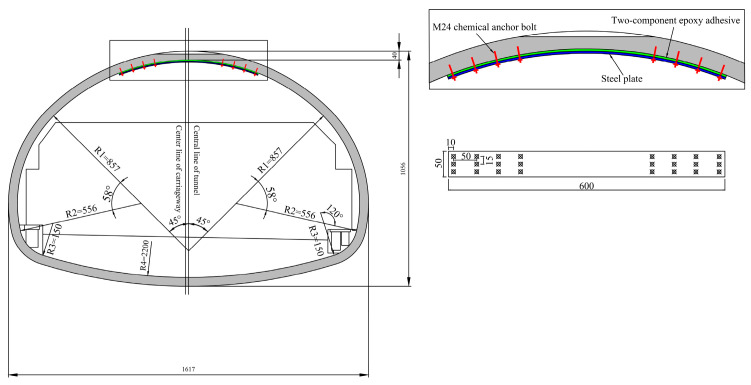
Steel plate reinforcement scheme for tunnel lining voids [22].

**Figure 2:**
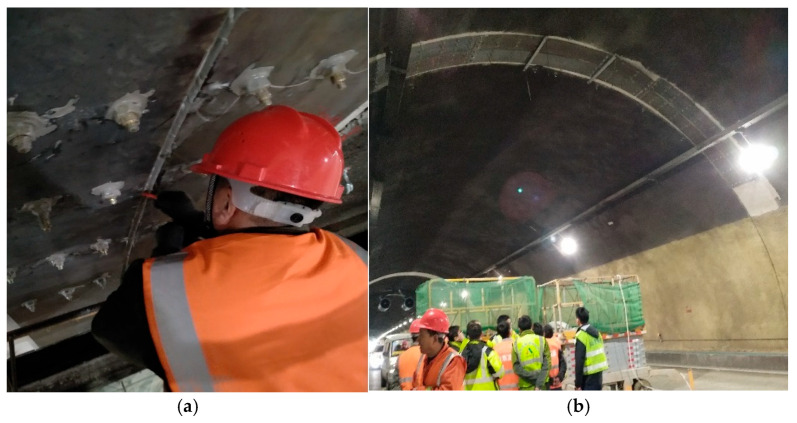
Installation of steel plate. (**a**) Installation process; (**b**) installation effect.

At the tunnel construction site, a steel plate reinforcement system was employed to address voids behind the lining, as illustrated in Figure 1. The field implementation of this steel plate reinforcement is shown in Figure 2. Within this reinforcement scheme, chemical anchor bolts were strategically positioned on both sides of the void-affected zone to fulfill two primary objectives: primarily to prevent damage to the lining structure with insufficient thickness within the void region, and secondly to ensure secure attachment of the steel plate against potential detachment through bilateral anchoring. Steel plates are bonded to the lining surface using epoxy adhesive. Prior to steel plate installation, the concrete surface should be cleaned and roughened. This process removes surface water and stains while increasing the contact area between the lining surface and the epoxy adhesive, ultimately enhancing bonding strength.

However, the current structural configuration exhibits the following limitations: (1) It cannot precisely determine the void extent compromises the accurate positioning of chemical anchor bolts. (2) Although the absence of chemical anchor bolts within the void area avoids further damage to the thinned lining in this zone, it results in inadequate rigid fixation of the steel plate. This deficiency increases the risk of plate-lining separation during deformation due to insufficient anchoring constraints. To address these issues, numerical simulations were conducted to investigate the reinforcement mechanisms of the steel plate system, with the aim of optimizing the design strategy for enhanced structural performance.

## 3. Analysis of Steel Plate Reinforcement Mechanism

### 3.1. Optimization of Numerical Simulation Methods for Concrete Damage

Current numerical simulation methods for characterizing concrete damage mainly include the Concrete Damage Plasticity (CDP) model [27,28,29], Extended Finite Element Method (XFEM) [30,31,32], Discrete Element Method (DEM) [33,34,35], and Phase Field method [36,37,38]. Studies in the literature indicate that CDP and XFEM are the most widely adopted approaches in damage analysis of concrete structures, yet they exhibit significant discrepancies in computational resource consumption and result visualization.

Based on the numerical model of locally delaminated reinforced concrete lining and its validation experiments established in reference [21], this study compared the numerical simulation effects of the concrete damage plasticity (CDP) model and the extended finite element method (XFEM). The applicability of the two methods was comprehensively evaluated, and the numerical simulation method more suitable for this working condition was selected.

**Figure 3:**
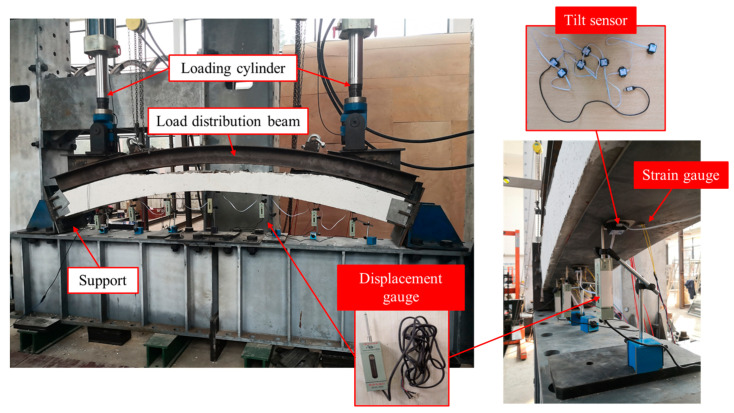
Loading device and measuring device [21].

**Figure 4:**
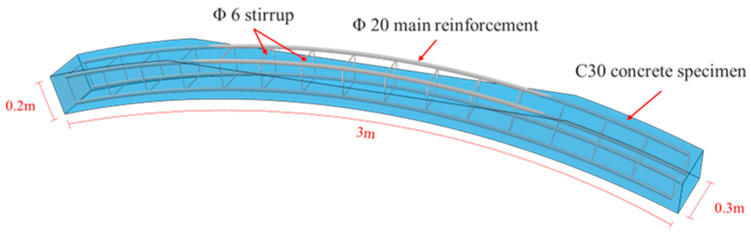
Specimen size.

As illustrated in Figure 3, the experiment utilized a static loading system with a maximum capacity of 500 kN. The specimen was fixed between its supports, with specific dimensions detailed in Figure 4. 7 measurement points were arranged on the lower surface of the specimen, located at the mid-span, at the edges of the openings, and at the midpoints between these aforementioned locations. Each measurement point was equipped with both a tilt sensor and a displacement sensor.

Numerical simulations were conducted using the Abaqus 2020 software package, employing both its built-in Concrete Damaged Plasticity (CDP) model and the Extended Finite Element Method (XFEM) model. Concrete was modeled using C3D8R elements (8-node linear hexahedral elements with hourglass control). Reinforcement bars and chemical anchor bolts were modeled using B32 elements (3-node quadratic beam elements). Steel plates were modeled using S4R elements (4-node reduced-integration shell elements with hourglass control). The concrete used in the specimens is grade C30. The elastic modulus is 3 × 10^4^ MPa. The Poisson’s ratio is 0.2, and the density is 2850 kg/m^3^. The steel reinforcement material adopts HRB335 grade, and its constitutive relationship is simulated using a bilinear model that accounts for the post-yield hardening stage. The material has a density of 7850 kg/m^3^, a Poisson’s ratio of 0.3, and an elastic modulus of 200 GPa. The mechanical performance parameters are as follows: standard yield strength *f_yk_* = 335 MPa, standard ultimate strength *f_stk_* = 455 MPa. The damage parameters for the CDP model were determined based on the stress–strain curve of C30 concrete specified in the Chinese National Standard for concrete design (GB50010-2010 [39]), calculated using Najar’s theory. In the XFEM model, the concrete material employed a maximum principal stress damage criterion, with the cracking stress threshold set to 20.1 MPa. Damage evolution followed a displacement-based cracking criterion, with the critical crack opening displacement specified as 0.0003 m [21].

**Figure 5:**
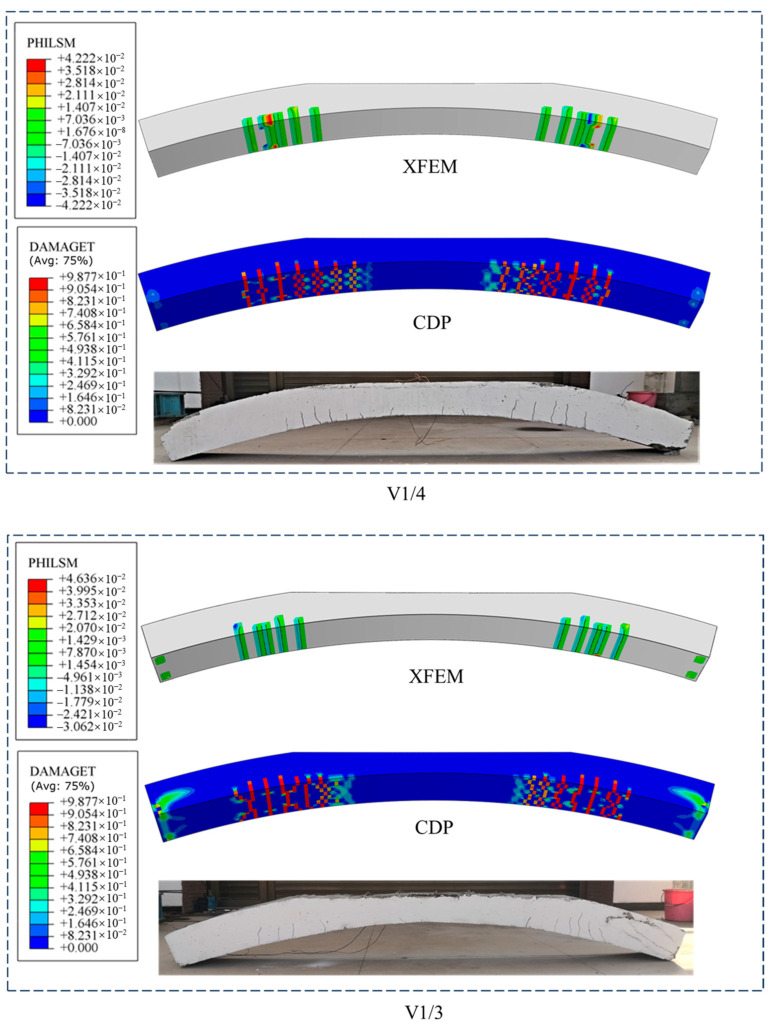
Comparison of simulation and experimental results.

The cracking conditions of each working condition under a load of 500 kN are compared with the test results as shown in Figure 5. The cracking areas of the debonded lining are mainly located at the debonding boundaries. The number of cracks is basically symmetrically distributed. From an overall perspective, the XFEM model provides a more intuitive display of cracking. However, it has a higher computational cost. When the model is large, the calculation time is long and unstable. It has high requirements for the mesh. When the mesh is fine, the calculation is difficult. Moreover, it is prone to errors when cracks intersect during the calculation. Since the plastic stage of concrete is not considered, the stress calculation results differ significantly from the actual situation. The CDP model shows the position of the crack by means of the damage parameters of the element. It has better adaptability to the mesh and higher computational efficiency, making it more suitable for large model calculations. In addition, it can be found from the simulation results of V1/3 that the CDP model accurately simulates the damage at the support, indicating that the CDP model is more precise in simulating concrete damage.

**Figure 6:**
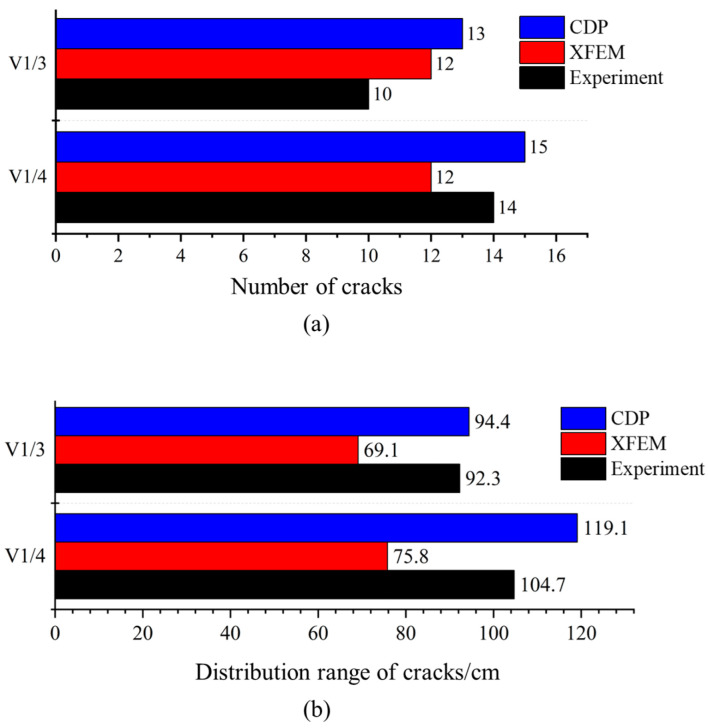
Comparison of crack conditions. (**a**) Number of cracks; (**b**) distribution range of cracks.

The number of cracks is also an important indicator of the accuracy of damage simulation methods. The closer the simulated crack number is to the experimental results, the better the simulation performance. As shown in Figure 6, the comparison of the number of side cracks under the two conditions indicates that both methods are relatively close to the experimental results in terms of crack quantity. However, under both conditions, the CDP model exhibits a slightly higher number of side cracks than observed in the experimental conditions, suggesting that the CDP model may have an advantage in capturing less severe cracking. In terms of crack distribution width, the CDP model is more accurate than the XFEM model, with a distribution width that more closely matches the experimental results, whereas the XFEM model shows a significantly narrower crack distribution width compared to the experimental findings.

**Figure 7:**
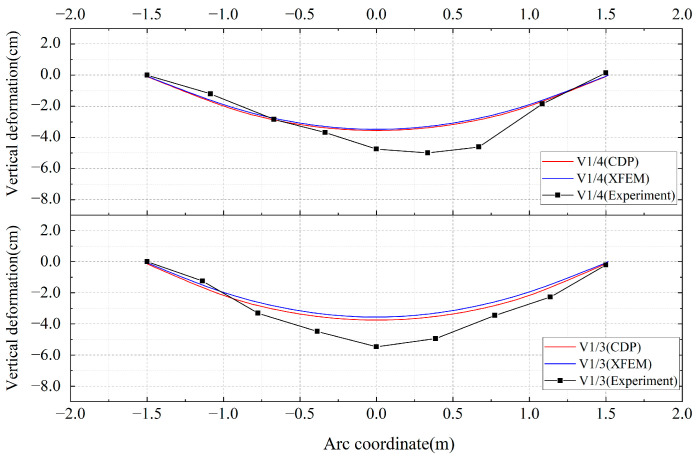
Comparison of vertical deformation in lining bottom.

The response of the simulations to the deformation of the lower surface can also indicate the precision of the simulation methods. Figure 7 presents a comparison of the vertical deformation of the lower surface between the two simulation methods and the experimental results. The results show that the predicted values from both simulation methods are lower than the experimental values. This discrepancy may be attributed to factors such as the precision of model fabrication during the experimental process, which are difficult to control precisely and may lead to larger experimental results. However, since the CDP model accounts for the effect of concrete plasticity on stiffness, it predicts a larger vertical deformation of the lower surface compared to the XFEM model, which is closer to the experimental data, thereby demonstrating higher simulation accuracy.

Considering all factors, the CDP model is deemed more appropriate for the calculation of the overall debonded lining model.

### 3.2. Numerical Simulation of the Strengthening Mechanism of Steel Plate Reinforcement

#### 3.2.1. Model Settings and Simulation Methods

**Figure 8:**
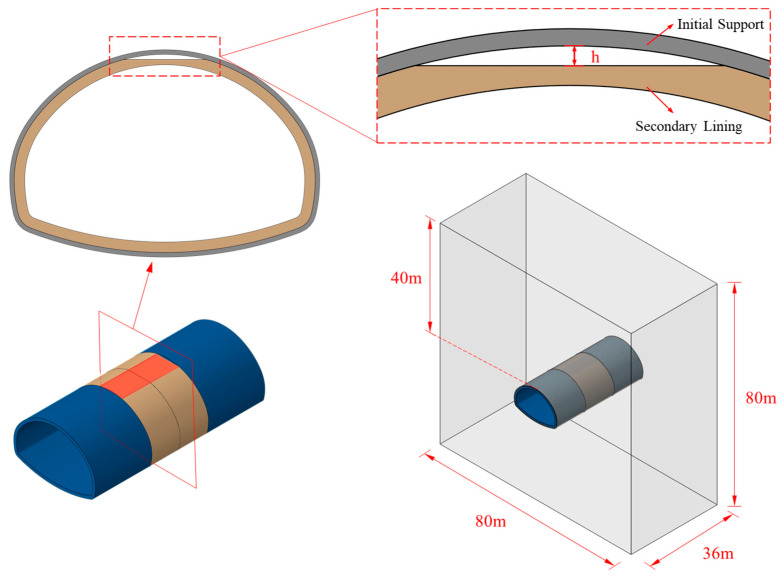
Model design [40].

Through the comparison of methods in the previous section, this study employs the Concrete Damage Plasticity (CDP) model to construct a three-dimensional numerical model for the overall steel plate reinforcement of tunnel lining with detached lining. The simulation is conducted using Abaqus. The geometric dimensions of the model are referenced from the data in the reference [40], with a focus on parameter modification of the key construction of steel plate reinforcement for the detached lining. As shown in Figure 8, in the numerical model, the designed thickness of the primary support layer is 28 cm, and the thickness of the secondary lining structure is 60 cm. The calculation adopts the ground structure method, with particular consideration of the dynamic loading process of the lining structure after the balance of ground stress. In terms of boundary condition settings: the bottom boundary of the model uses fixed constraints, and the side boundaries are set with normal displacement constraints to simulate the actual surrounding rock constraint state.

**Figure 9:**
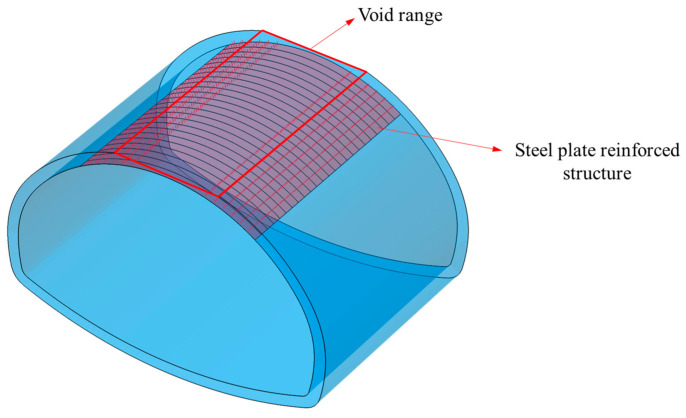
Steel plate reinforcement scheme.

As shown in Figure 9, 24 steel plates are continuously arranged to reinforce the vault void area of the secondary lining. Each plate measures 10 m in length, 0.5 m in width, and 10 mm in thickness, modeled using shell elements. On each side of the void area, M24 chemical anchors are arranged in 3 rows and 6 columns (totaling 18 anchors per side; 36 anchors in total), modeled using beam elements. The adhesive layer between the steel plate and the lining is simulated using cohesive contact, and the viscosity of the structural adhesive is expressed using the “relative separation displacement–force” relationship between the steel plate surface and the lining surface. The normal tensile strength of the adhesive layer is 30 MPa, and the tangential tensile strength is 20 MPa.

#### 3.2.2. Materials

The primary support is constructed using C25 concrete, while the secondary lining is modeled with C30 concrete. The upper half of the secondary lining is represented by a concrete material incorporating plasticity and damage characteristics, whereas the lower half is simulated as an elastic material. The material properties, including an elastic modulus of 3 × 10^4^ MPa, a Poisson’s ratio of 0.2, and a density of 2850 kg/m^3^, are specified in Table 1.

The damage factor parameters required for simulation are calculated through the Gauss numerical integration method based on Najar’s damage factor calculation theory [41]. As shown in Table 2 and Table 3, they are, respectively, the compressive and tensile damage factors of C30 concrete obtained.

#### 3.2.3. Simulation Results

**Figure 10:**
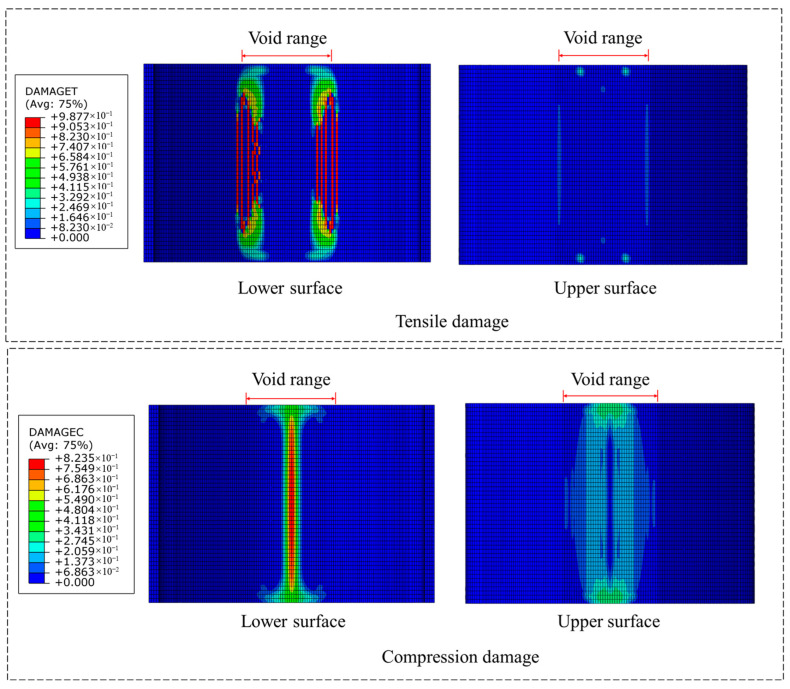
Damage situation under unreinforced working conditions.

**Figure 11:**
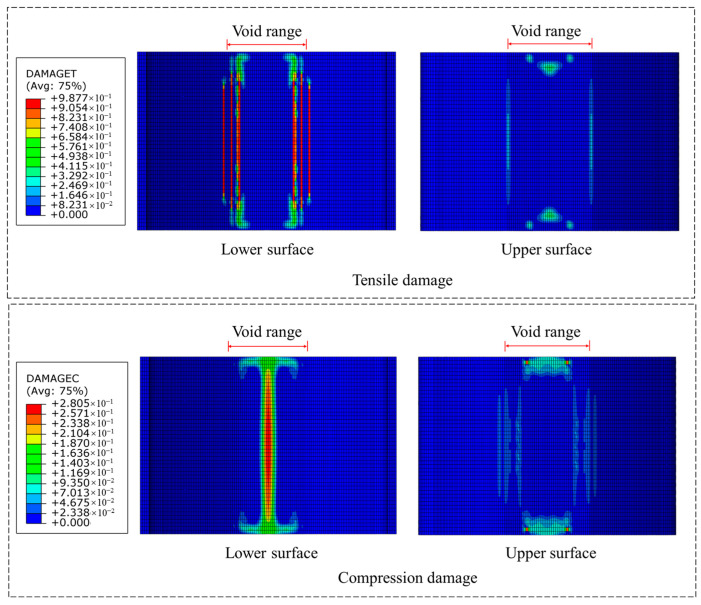
Damage situation using chemical anchor bolts alone.

**Figure 12:**
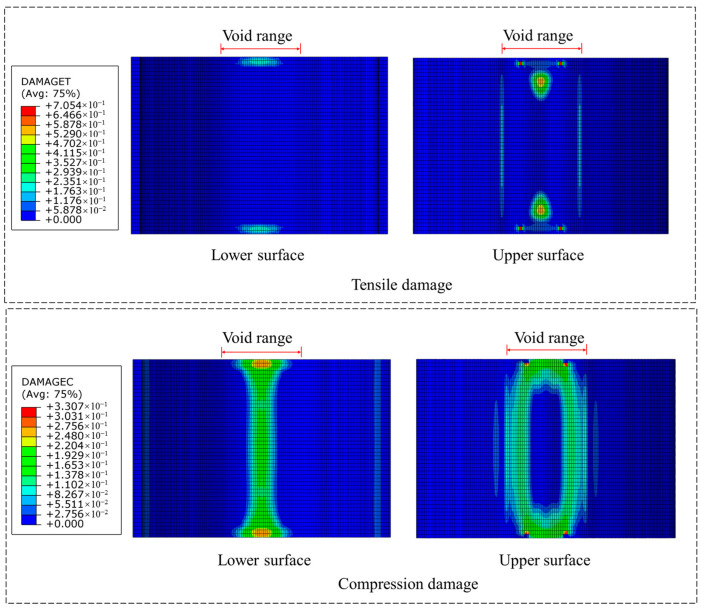
Damage situation using structural adhesive alone.

**Figure 13:**
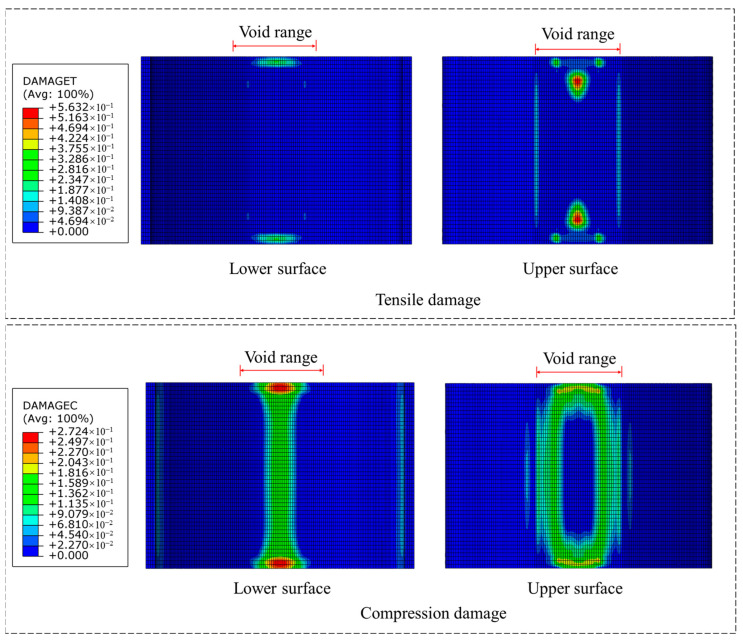
Damage situation using structural adhesive and chemical anchor bolt combination.

Figure 10, Figure 11, Figure 12 and Figure 13, respectively, show the damage conditions of tunnel linings under unreinforced conditions and three different reinforcement scenarios. As can be seen from Figure 10, in the unreinforced condition, multiple distinct longitudinal tensile cracks appear near the boundary of the lower surface void. This indicates that the concrete tensile strength has been exceeded at the void boundary of the lower surface, resulting in extensive tensile damage. Meanwhile, significant compressive damage is observed at the intrados of the vault crown, suggesting that the compressive stress in this area has surpassed the concrete compressive strength.

When chemical anchor bolts reinforcement alone is applied (Figure 11), the longitudinal cracks at the void boundary of the lower surface show notable restraint with significantly reduced quantity. The compressive damage factor at the vault crown decreases substantially from 0.82 in the unreinforced condition to 0.28, indicating effective control of compressive damage.

As demonstrated in Figure 12 for structural adhesive reinforcement alone, the cracking at the void boundary of the lower surface disappears completely, showing excellent reinforcement effectiveness against boundary cracking. Concurrently, the compressive damage at the vault crown is also significantly mitigated, demonstrating ideal reinforcement outcomes.

Figure 13 illustrates the combined use of structural adhesive and chemical anchor bolts. In this composite reinforcement scenario, tensile damage at the void boundary of the lower surface is completely eliminated, and the compressive damage factor at the vault crown remains below the threshold for significant damage. Notably, in both scenarios involving structural adhesive, although large-scale damage is prevented, localized minor damage areas emerge near the lateral construction joints. This phenomenon may be attributed to excessive constraint from the reinforcement system during deformation-induced compression by adjacent lining segments. In practical applications, this issue could be addressed by reserving appropriate lengths at lateral construction joints during steel plate installation.

## 4. Simulation of Optimizing the Spacing Between Steel Plate Layouts

**Figure 14:**
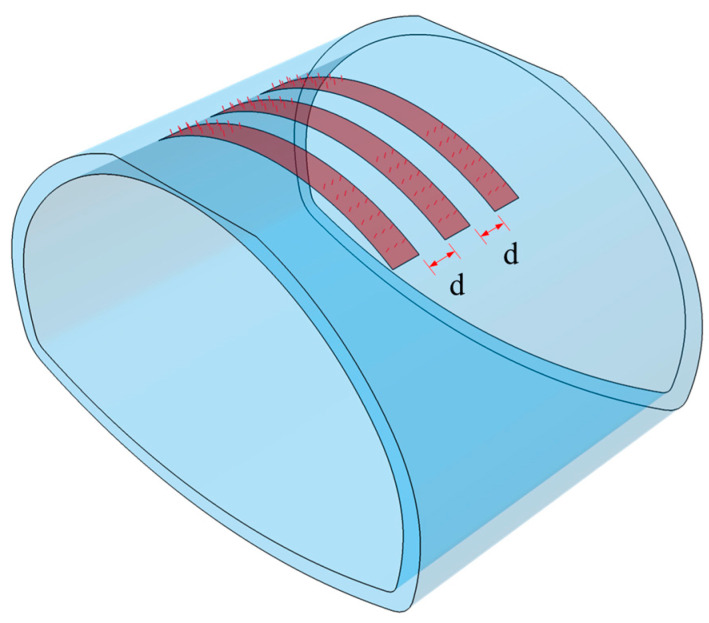
Schematic diagram of steel plate position and spacing.

For existing steel plate reinforcement structures, optimization initially focuses on the spacing of the steel plates. When reinforcing linings with small void heights, rational arrangement of steel plate spacing can not only effectively reduce reinforcement costs and enhance construction efficiency but also ensure uniform distribution of structural adhesive, thereby improving the reinforcement effectiveness. Based on this, as shown in Figure 14, three computational cases with steel plate spacing ***d*** of 0.25 m, 0.5 m, and 1 m were established under the condition of a steel plate width of 0.5 m. By modeling and analyzing the influence of different spacings on the reinforcement effectiveness of the lining, the optimal maximum steel plate spacing that ensures reinforcement effectiveness can be determined.

**Figure 15:**
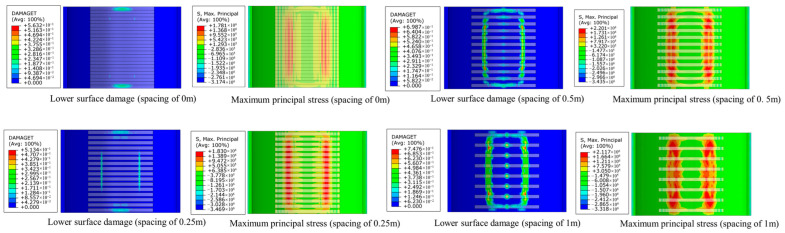
Surface damage and maximum principal stress of void lining reinforced with steel plates of different spacing.

Figure 15 presents the tensile damage and corresponding maximum principal stress contour maps on the lower surface of the lining under various steel plate spacings. As shown in the figure, when the steel plate spacing is 0, the reinforcement effect on the lower surface of the lining is optimal, with a maximum damage factor value of 0.289 and a maximum principal stress of 1.78 MPa. The overall stress level remains below the tensile strength of concrete, with no significant damage observed. As the steel plate spacing increases to 0.25 m, the maximum damage factor value rises to 0.513, and the maximum principal stress increases slightly to 1.83 MPa. The increase in the maximum principal stress is minimal, and the reinforcement effect remains largely unchanged. When the spacing is further increased to 0.5 m, tensile stress zones exceeding the tensile strength of concrete begin to appear, with the maximum damage factor increasing to 0.699 and the maximum principal stress rising to 2.20 MPa. This exceeds the tensile strength of concrete, leading to the onset of tensile damage and a reduction in reinforcement effectiveness. When the steel plate spacing is further increased to 1 m, the constraint force of the steel plate on the lining weakens, resulting in a further increase in the damage factor to 0.748. The concrete enters the plastic region, with a maximum principal stress of 2.12 MPa. The reinforcement effectiveness continues to deteriorate and is no longer able to meet the requirements.

**Figure 16:**
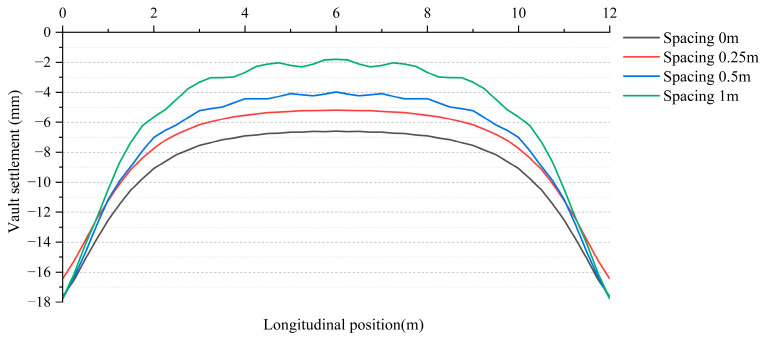
Vault settlement distribution of steel plate-reinforced linings with different spacings.

Figure 16 illustrates the influence of different steel plate spacings on crown settlement. The curves in the figure represent the settlement conditions for steel plate spacings of 0 m, 0.25 m, 0.5 m, and 1 m, respectively. It can be observed that as the steel plate spacing increases, the relative uplift amplitude of the crown also correspondingly increases. When the steel plate spacing is 0 m, the relative uplift value of the crown is the smallest at 11.0 mm, with the flattest curve. This indicates that in the absence of spacing, the steel plate provides the best control over the relative deformation of the crown, effectively restricting the uneven settlement of the crown. As the spacing increases to 0.25 m, the relative uplift value of the crown increases to 11.3 mm, but the increase is minimal and remains essentially consistent with the case of 0 m spacing. This suggests that even with a small spacing, the steel plate can still provide a relatively good deformation control effect. When the steel plate spacing further increases to 0.5 m, the relative uplift value of the crown rises to 13.8 mm, and the curve exhibits more pronounced relative fluctuations. This indicates that as the spacing increases, the ability of the steel plate to restrict deformation of the lining begins to weaken. When the steel plate spacing reaches 1 m, the relative uplift value is the largest at 16.0 mm, with the most significant curve fluctuations. This suggests that with a larger spacing, the reinforcement effect of the steel plate on the crown is greatly reduced, resulting in poor settlement control.

The comprehensive results of this study demonstrate that the optimization of steel plate spacing is crucial for enhancing the reinforcement effectiveness of lining structures. The steel plate spacing not only affects the magnitude and uniformity of stress distribution but also directly influences the overall performance and durability of the reinforced structure. Through systematic numerical simulations, it has been determined that a steel plate spacing of 0.25 m can maximize the restoration of the load-bearing capacity of void linings while maintaining the lowest reinforcement cost and workload, making it the optimal spacing for voids. In practical reinforcement projects, when the void area is large or the void depth is significant, it is recommended to use continuous steel plate reinforcement without spacing for the lining structure. Conversely, for smaller-scale or shallower voids, a steel plate spacing of 0.25 m is recommended to reduce the reinforcement workload and cost while ensuring effective reinforcement.

## 5. Optimization of Steel Plate Structure

A comprehensive comparison of the three reinforcement schemes reveals that the reinforcement effectiveness of using structural adhesive alone is only slightly inferior to that of the combined use of chemical anchor bolts and structural adhesive. In contrast, reinforcement using chemical anchor bolts alone exhibits relatively weaker effectiveness, indicating that structural adhesive plays a dominant role in steel plate reinforcement. However, despite the primary reinforcement function of structural adhesive, the role of chemical anchor bolts should not be overlooked. In particular, chemical anchor bolts can provide essential fixation when structural adhesive fails, thereby effectively preventing the detachment of steel plates and ensuring structural safety. Localized simulation models, however, show that an excessive number of chemical anchor bolts can cause a certain degree of localized damage to the lower surface of the lining at the chemical anchor bolt connections.

**Figure 17:**
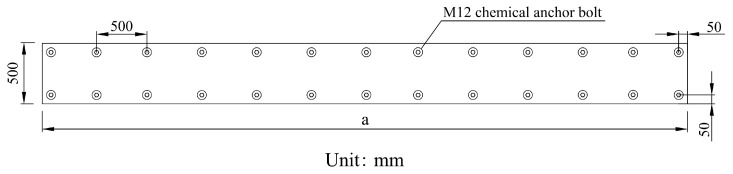
Optimized steel plate structure.

Therefore, optimization of the steel plate reinforcement structure is recommended. As shown in Figure 17, structural adhesive is employed to bear the primary reinforcement load, while redundant chemical anchor bolts are removed. Only M12 small-diameter chemical anchor bolts, spaced at 300 mm, are arranged along the edges of the steel plate for fixation, with a 50 mm distance maintained between the chemical anchor bolts and the steel plate edges. The length ‘a’ of the steel plate is determined by the scope of field reinforcement and the practicality of construction. Through this optimized design, for voids suitable for steel plate reinforcement, chemical anchor bolts no longer cause penetrating damage to the lining within the void range. This approach not only reduces local damage to the lower surface of the void lining caused by large-diameter chemical anchor bolts but also decreases the workload, lowers costs, and enhances reinforcement efficiency.

**Figure 18:**
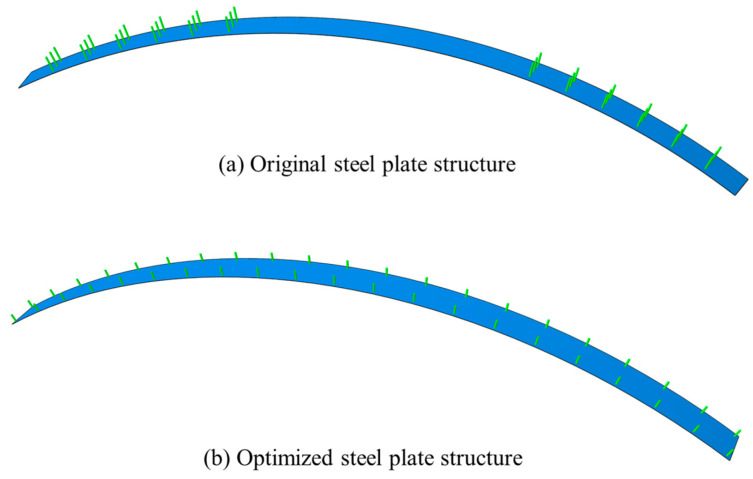
Comparison of steel plate structures.

As depicted in Figure 18, to validate the reinforcement efficacy of the optimized steel plate structure, the original steel plate structure with a spacing of 0.25 m was optimized. A numerical model of the optimized structure was subsequently established, and the stress and damage conditions of the two structures were comparatively analyzed.

**Figure 19:**
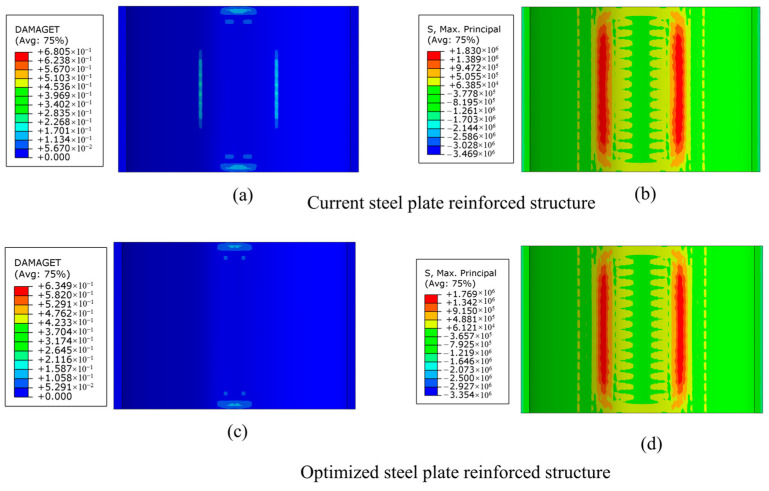
Comparison diagram of reinforcement effect of steel plate structure. (**a**) Tensile damage to the original steel plate structure; (**b**) maximum principal stress of the original steel plate structure; (**c**) tensile damage of optimized steel plate structure; (**d**) maximum principal stress of optimized steel plate structure.

Figure 19a,c show a comparison of tensile damage for the two structures. In the original structure, the peak damage value reaches 0.68, and there are regions with relatively high damage values near the void boundaries, indicating that the material in these areas is close to its load-bearing limit. In the optimized structure, the peak damage value is reduced to 0.63, and the area of high-damage regions is significantly decreased, particularly with no significant high-damage regions appearing on the two sides of the void where stress is higher.

Figure 19b,d present a comparison of the stress distribution on the lower surface of the lining after reinforcement using two different steel plate reinforcement structures. In the original structure, the maximum principal stress ranges from +1.830 × 10^6^ MPa to −3.469 × 10^6^ MPa, with the maximum tensile stress reaching 1.830 × 10^6^ MPa. In the optimized structure, the maximum tensile stress is reduced to 1.769 × 10^6^ MPa, and the maximum compressive stress is lowered to −3.354 × 10^6^ MPa. The overall stress distribution is more balanced, with both the maximum tensile and compressive stresses being decreased.

The simulation results reveal that, in comparison with the original structure, the optimized structure, which features reduced chemical anchor bolt dimensions and optimized distribution, has achieved an enhanced reinforcement effect. While maintaining the overall crack resistance, the optimized structure has managed to reduce local damage and the consumption of anchor bolts. Consequently, the reinforcement cost has been lowered, the construction difficulty has been mitigated, the construction speed has been accelerated, the structural durability has been improved, and the impact on tunnel operation has been minimized.

## 6. Discussion

Current methods for treating tunnel lining detachment—including bonding carbon fiber-reinforced polymer (CFRP) sheets, steel plate bonding, constructing an umbrella arch, creating skylight openings, or complete demolition and reconstruction—each present significant limitations. CFRP, while lightweight, is prone to brittle interfacial debonding or fracture at existing cracks, failing to provide robust crack control or effectively redistribute stresses caused by voids. Umbrella arch reinforcement, though structurally sound, entails high costs, extended construction time, and significant intrusion into the tunnel clearance envelope. Skylight openings or demolition severely compromise the structural integrity of the original lining, drastically alter stress distributions, pose major construction safety challenges, and cause substantial traffic disruption. In contrast, steel plate bonding provides superior tensile strength and ductility, effectively transferring loads away from void-affected areas to mitigate localized stress concentrations. Crucially, it offers minimal clearance intrusion, relatively simple and rapid installation, lower overall cost, and significantly reduced operational disruption, presenting an optimal balance of structural performance and practical feasibility for void remediation.

Compared with the existing research literature on steel plate reinforcement [42,43,44], the primary advantage of this paper is to realize the simulation experiment of full-scale lining structure and investigate a wider range of working conditions. However, the existing literature mostly relies on physical tests, and generally uses a scaled model or the lining model with limited longitudinal length, which has a significant size effect, and the working conditions that can be studied are relatively limited. In addition, the existing research mainly focuses on the evaluation of the reinforcement effect, with less in-depth analysis of the reinforcement mechanism; the research on the key parameter of longitudinal spacing of steel plate is rare. The simulation case study presented in this paper focuses on optimizing steel plate reinforcement schemes for lining sections of this specific size, considering only geological influences to demonstrate the feasibility of the research methodology. When factors such as variations in soil parameters, groundwater, or seismic activity are present, converting these factors into additional lining loads may yield divergent results.

## 7. Conclusions

This study employs numerical simulations to investigate the reinforcement mechanisms of steel plate reinforcement for lining debonding, based on existing reinforcement schemes. The study optimizes the steel plate reinforcement schemes from two aspects: steel plate spacing and reinforcement structure. The main conclusions can be summarized as follows:

(1) A comparative analysis of existing debonding reinforcement methods revealed that steel plate reinforcement exhibits good adaptability for moderate debonding and offers significant advantages due to its shorter construction time.
(2) This study investigated the reinforcement mechanism of steel plate reinforcement for void lining. The reinforcement effects of adhesive and chemical anchor bolts, both individually and in combination, were examined. The results indicated that adhesive plays a primary role in stress transfer within the reinforced structure, while the contribution of chemical anchor bolts to stress transfer is relatively limited. Chemical anchors mainly perform the role of fixing the steel plate.
(3) Investigations into steel plate spacing demonstrated that a longitudinal spacing of 0.25 m between steel plates can effectively balance reinforcement effectiveness and cost reduction. Compared with the working condition with the spacing of 0, the maximum principal stress of the steel plate reinforced void lining increases from 1.78 MPa to 1.83 MPa at the spacing of 0.25 m, which remains basically unchanged. At the same time, the damage to the lining concrete has not increased, which can reduce the number of steel plates under the premise of maintaining the reinforcement effect. Additionally, maintaining a certain interval between steel plates facilitates the densification of the adhesive layer during injection, thereby enhancing the overall reinforcement quality.
(4) Further study on the reinforcement mechanism shows that the chemical anchor bolt not only has a limited reinforcement effect, but also causes surface damage to the lining during installation. Consequently, the steel plate reinforcement structure was optimized by reconfiguring the layout of chemical anchor bolts. The large-size chemical anchors of the steel plate were canceled, and only the small-size chemical anchors were arranged at the edge of the steel plate. Numerical simulations were conducted to compare the reinforcement effects of the original and optimized steel plate reinforcement structures. The optimized structure was found to achieve comparable reinforcement effectiveness while significantly reducing the number of chemical anchor bolts required, thereby lowering reinforcement costs and improving efficiency.

## Figures and Tables

**Table 1:** Detailed information of the material parameters.

	Density (kg·m^−3^)	Elastic Modulus (GPa)	Poisson’s Ratio	Internal Friction Angle (°)	Cohesion (kPa)
Surrounding rock	2400	1.5	0.3	30	700
Initial support	2400	28	0.2		
Secondary lining	2850	30	0.2		

**Table 2:** C30 concrete compressive damage factor.

Compressive Stress/MPa	Compressive Inelastic Strain	Concrete Compressive Damage Factor *d_c_*
0	0	0
15.45	0.000732	0
18.48	0.001027	0.153
19.93	0.001321	0.233
20.10	0.001468	0.272
19.61	0.001762	0.347
15.88	0.002646	0.533
12.49	0.003529	0.659
10.07	0.004411	0.742
7.5	0.005883	0.826
4.89	0.009227	0.911

**Table 3:** C30 concrete tensile damage factor.

Tensile Stress/MPa	Tensile Cracking Strain	Concrete Tensile Damage Factor *d_t_*
0	0	0
2.01	0	0
0.66	0.00035	0.153
0.42	0.000647	0.233
0.32	0.000937	0.272
0.23	0.001513	0.347
0.18	0.002087	0.533
0.15	0.00266	0.659
0.13	0.003327	0.742

## Data Availability

The original contributions presented in this study are included in the article. Further inquiries can be directed to the corresponding authors.
